# Ratio of extracellular water and body cell mass is associated with coronary artery calcification in peritoneal dialysis patients

**DOI:** 10.3389/fmed.2026.1895552

**Published:** 2026-09-21

**Authors:** Ying Liu, Yan Liang, Yuan Feng, Jing Liu, Qiuyuan Shao, Cheng Wan, Jiaqian Song, Rining Tang, Sixiu Liu, Chunming Jiang

**Affiliations:** Department of Nephrology, Nanjing Drum Tower Hospital, The Affiliated Hospital of Nanjing University Medical School, Nanjing, Jiangsu, China

**Keywords:** body cell mass, body composition, coronary artery calcification, extracellular water, peritoneal dialysis

## Abstract

**Background:**

Coronary artery calcification (CAC) is seen in most patients on dialysis and is strongly associated with cardiovascular mortality. The extracellular water to body cell mass ratio (ECW/BCM), as an indicator of nutritional status and fluid overload, is closely associated with the prognosis of dialysis patients. However, its relationship with CAC at peritoneal dialysis (PD) initiation remains unclear.

**Methods:**

Incident PD patients in our hospital between 1 January 2022 and 31 December 2024 were included in this retrospective study. CAC was assessed by multislice spiral computed tomography. ECW and BCM were measured by bioelectrical impedance analysis (BIA). Patients were divided into three groups according to ECW/ BCM tertiles. Logistic regression analyse were performed to explore the association between ECW/BCM with CAC. Receiver operating characteristic (ROC) curves were used to evaluate the predictive value of ECW/BCM for CAC.

**Results:**

A total of 197 PD patients were recruited and the prevalence of CAC was 53.81%. Patients with a higher ECW/BCM ratio had a higher prevalence of CAC and CACS level. The multivariate logistic regression identified ECW/BCM as an independent risk factor for CAC [per 0.1 increase of ECW/BCM, odds ratio 1.350, 95% confidence interval (CI) 1.089–1.674, *p* = 0.006]. The area under the receiver operating characteristic curve (AUC) for predicting CAC further increased to 0.869 (95%CI 0.818–0.920) after ECW/BCM was added to conventional factors.

**Conclusion:**

ECW/BCM was independently associated with the presence of CAC in the early stages of PD.

## Introduction

Cardiovascular events (CVEs) are one of the leading causes of death in patients on peritoneal dialysis (PD). The prevalence of vascular calcification in dialysis patients is significantly higher than in the general population (1), and most patients initiating dialysis already have signs of advanced atherosclerosis (2). The presence of vascular calcification were closely related to CVEs and all-cause mortality in PD patients (3, 4). Therefore, early identification of risk factors associated with coronary artery calcification (CAC) is instrumental for the intervention of CVEs in PD patients.

Fluid overload and malnutrition are common complications in PD patients. Previous studies have shown that overhydration is associated with cardiovascular morbidity (5). Furthermore, a recent study by Park et al. (6) found that extracellular fluid status is significantly associated with CAC. Malnutrition has also been shown to be associated with atherosclerosis and cardiovascular disease in dialysis patients (7).

Bioimpedance analysis (BIA) has been recognized as a simple and non-invasive method for analysing body composition and hydration status (8). BIA-derived extracellular water/body cell mass ratio (ECW/BCM) is a sensitive index reflecting fluid overload and nutritional status in dialysis patients (9, 10). Previous studies have indicated that ECW/BCM is a potential predictor of mortality in dialysis patients (10). However, it is unclear whether ECW/BCM is linked to CAC in patients beginning PD. The aim of this study is to analyze the correlation between ECW/BCM and CAC in incident PD.

## Methods

### Study design and participants

This was a single-center retrospective study that included patients who underwent their first PD catheter placement surgery and initiated PD treatment at Nanjing Drum Tower Hospital between 1 January 2022 and 31 December 2024. Patients who aged ≥18 years and underwent coronary CT and BIA examinations within the first 3 months after PD initiation were analyzed. Patients were excluded if they met the following criteria: (1) pacemaker implantation or severe limb defects that could not be examined by BIA; (2) incomplete clinical data; (3) malignant tumors or severe infections.

### Body composition measurement

Body composition was measured using BIA. All patients were analyzed by trained technicians using the portable body composition monitor (Fresenius Medical Care, Bad Homburg, Germany). All BIA measurements were performed in the supine position after complete drainage of peritoneal dialysate. The main parameters of interest included extracellular water (ECW), body cell mass (BCM), lean tissue index (LTI) and fat tissue index (FTI). The ECW/BCM ratio was calculated ECW by BCM.

### Evaluation of CAC

CAC was evaluated using multislice spiral computed tomography and recorded as the Agatston score (11). CACS of left coronary artery trunk, circumflex branch of left coronary artery, descending branch of left coronary artery and right coronary artery were calculated, respectively. The scores were then summed to calculate the total CACS and was expressed in modified Agatston units (AU). According to the CACS, patients with a CACS of 0 were classified into the non-CAC group and the others into the CAC group.

### Clinical data collection

The clinical data collected during the first CAC assessment serves as the baseline data. Data collection included demographics, cause of end-stage renal disease, comorbidities, laboratory data (hemoglobin, albumin, calcium, phosphorus, cholesterol, etc.), dialysis adequacy parameters and medication use.

### Statistical analysis

Data were analysed using SPSS version 24.0, GraphPad Prism version 8.1.0 and R language version 4.6.0. Continuous variables that conformed to a normal distribution are expressed as mean ± standard deviation (x ± s), and Intergroup comparisons were performed using independent-sample Student’s *t*-tests or analysis of One-way ANOVA. Continuous variables that were not normally distributed are expressed as medians (interquartile range [IQR]), and Mann–Whitney U test or Kruskal–Wallis *H* test were used for comparisons. Categorical variables were expressed as percentages and Chi-squared test or Fisher’s exact test were used for comparisons between groups. Spearman correlation analysis was performed to identify the relationship between ECW/BCM and clinical data. A logistic regression model was used to evaluate the independent association of ECW/BCM with CAC. The predictive value of ECW/BCM for CAC was determined using receiver operating characteristic (ROC) curves, net reclassification improvement (NRI), and integrated discrimination improvement (IDI). Sensitivity analysis was performed using linear regression models. A *p* value <0.05 was considered statistically significant.

## Results

### Baseline characteristics

A total of 197 patients were recruited (118 mean, 59.90%), with a median age of 52.00 (37.50, 63.00) years. Chronic glomerulonephritis is the primary cause of end-stage renal disease (ESRD) (90/197, 45.69%), and 73 patients (37.06%) with diabetes mellitus (DM). The median CACS was 3.38 (0, 207.98), and the prevalence of CAC was 53.81%. The median ECW/BCM was 0.79 (0.68, 0.96).

ECW/BCM was categorized into three groups based on tertiles: low (<0.71), middle (0.71–0.90), and high (>0.90). Patients in the high ECW/BCM group were older and had a higher prevalence of female, DM and heart disease. They also had higher levels of BMI, CRP, FTI and CACS. In addition, albumin, creatinine, urea nitrogen, phosphorus, iPTH, LTI and use of vitamin D receptor activator were significantly lower in the high ECW/BCM group (Table 1).

**Table 1:** Patient characteristics by ECW/BCM groups.

Factor	Total	Low (*n* = 65)	Middle (*n* = 67)	High (*n* = 65)	*p* value
Age (years)	52.00 (37.50, 63.00)	39.00 (33.00, 51.50)	55.50 (40.50, 65.00)	60.00 (51.00, 73.00)	<0.001
male (*n*/%)	118 (59.90)	44 (67.69)	43 (64.18)	31 (47.69)	0.045
History of DM (*n*/%)	73 (37.06)	8 (12.31)	30 (44.78)	35 (53.85)	<0.001
History of heart disease (*n*/%)	41 (20.81)	4 (6.15)	16 (23.88)	21 (32.31)	<0.001
BMI (kg/m^2^)	22.57 (19.95, 25.50)	21.60 (18.62, 23.95)	23.62 (20.94, 26.70)	23.00 (20.50, 25.50)	0.010
Hemoglobin (g/L)	98.00 (86.00, 110.00)	95.00 (85.50, 108.00)	97.00 (85.75, 110.00)	101.00 (86.00, 14.00)	0.640
Creatinine (umol/L)	686.00 (555.00, 921.00)	820.00 (655.00, 1084.00)	689.00 (567.75, 866.00)	582.00 (419.00, 766.00)	<0.001
Urea nitrogen (mmol/L)	21.10 (17.65, 25.05)	24.00 (19.80, 26.70)	20.85 (17.43, 24.65)	20.10 (16.60, 23.40)	0.006
Albumin (g/L)	32.14 ± 0.34	34.70 ± 0.50	32.66 ± 0.57	29.04 ± 0.50	<0.001
Cholesterol (mmol/L)	3.76 (3.11, 4.44)	3.76 (3.19, 4.20)	3.94 (3.14, 4.54)	3.62 (2.90, 4.31)	0.270
Triglyceride (mmol/L)	1.38 (0.93, 2.00)	1.31 (0.97, 1.94)	1.54 (1.05, 2.06)	1.34 (0.77, 2.03)	0.171
Calcium (mmol/L)	2.12 (2.00, 2.26)	2.15 (2.05, 2.30)	2.13 (1.98, 2.26)	2.09 (1.95, 2.21)	0.095
Phosphorus (mmol/L)	1.67 (1.40, 2.08)	1.82 (1.45, 2.22)	1.63 (1.44, 2.01)	1.57 (1.26, 1.96)	0.021
C-reactive protein (mg/L)	4.00 (2.60, 7.30)	3.40 (2.60, 4.75)	3.45 (2.18, 9.25)	5.10 (3.00, 9.60)	0.027
iPTH (pmol/L)	24.90 (14.08, 38.48)	31.60 (17.15, 42.84)	24.79 (13.88, 33.95)	19.13 (9.74, 37.25)	0.036
Total CCr (L/W/1.73 m^2^)	64.63 (50.32, 80.88)	64.63 (45.91, 78.22)	68.36 (52.06, 86.06)	62.59 (52.14, 80.82)	0.367
Total Kt/V	1.54 (1.29, 1.91)	1.48 (1.30, 1.79)	1.59 (1.34, 1.94)	1.54 (1.26, 1.95)	0.626
LTI (kg/m^2^)	13.45 ± 0.22	15.78 ± 0.34	13.87 ± 0.26	10.68 ± 0.24	<0.001
FTI (kg/m^2^)	8.30 (5.90, 11.65)	6.10 (3.45, 8.00)	8.60 (6.40, 11.80)	11.00 (8.20, 13.55)	<0.001
ECW/BCM	0.79 (0.68, 0.96)	0.64 (0.57, 0.68)	0.79 (0.75, 0.84)	1.05 (0.96, 1.27)	<0.001
Vitamin D receptor activator (*n*/%)	92 (46.70)	34 (52.31)	36 (53.73)	22 (33.85)	0.039
Calcium-based phosphate binder (*n*/%)	30 (15.31)	12 (18.46)	8 (11.94)	10 (15.38)	0.580
Statin (*n*/%)	52 (26.40)	12 (18.46)	19 (28.36)	21 (32.31)	0.182
CAC (*n*/%)	106 (53.81)	19 (29.23)	36 (53.73)	51 (78.46)	<0.001
Baseline CaCS	3.38 (0, 207.98)	0 (0, 4.36)	2.49 (0, 241.04)	96.53 (1.56, 511.81)	<0.001

DM, diabetes mellitus; BMI, body mass index; iPTH, intact parathyroid hormone; CCr, creatinine clearance rate; Kt/V: urea clearance.

### Association of clinical variables with ECW/BCM

Spearman correlation analysis showed that ECW/BCM was positively correlated with age (*r* = 0.506, *p* < 0.001), BMI (*r* = 0.110, *p* = 0.015), CACS (*r* = 0.475, *p* < 0.001), CRP and FTI (*r* = 0.551, *p* < 0.001). Statistically significant negative correlations were observed between ECW/BCM and creatinine (*r* = −0.383, *p* < 0.001), urea nitrogen (*r* = −0.264, *p* < 0.001), albumin (*r* = −0.522, *p* < 0.001), calcium (*r* = −0.160, *p* = 0.025), phosphorus (*r* = −0.230, *p* = 0.001), iPTH (*r* = −0.161, *p* = 0.024) and LTI (*r* = −0.551, *p* < 0.001) (Table 2).

**Table 2:** Correlation analysis of variables associated with ECW/BCM.

Factor	Spearman	
	*r*	*p* value
Age	0.506	<0.001
BMI	0.110	0.015
CACS	0.475	<0.001
Hemoglobin	0.096	0.182
Creatinine	−0.383	<0.001
Urea nitrogen	−0.264	<0.001
Albumin	−0.522	<0.001
Cholesterol	−0.051	0.475
Calcium	−0.160	0.025
Phosphorus	−0.230	0.001
C-reactive protein	0.219	0.002
iPTH	−0.161	0.024
LTI	−0.751	<0.001
FTI	0.551	<0.001

### Association between ECW/BCM and CAC

Univariate logistic regression showed that ECW/BCM was significantly associated with CAC. After adjusting for demographic characteristics, laboratory variables and medication history, ECW/BCM remained an independent risk factor for CAC (model 3 in Table 3; per 0.1 increase, OR 1.350, 95% CI 1.089–1.674, *p* = 0.006).

**Table 3:** Multivariable analysis of the association between ECW/BCM and CAC.

Factor	OR (95%CI)	*p* value
ECW/BCM (per 0.1 increase)	1.590 (1.333–1.896)	<0.001
Model 1	1.323 (1.073–1.630)	0.009
Model 2	1.323 (1.073–1.630)	0.009
Model 3	1.350 (1.089–1.674)	0.006

Model 1 adjusted for age, gender, history of DM and heart disease; Model 2 adjusted for all factors in model 1 plus albumin, cholesterol, calcium and phosphorus; Model 3 adjusted for all factors in model 2 plus use of vitamin D receptor activator and statin.

### Analysis of subgroups

To assess the robustness of the primary outcome, we performed subgroup analyses to explore the influence of factors such as age, sex, and diabetes status on the association. As shown in Figure 1, ECW/BCM was significantly higher in the CAC group than in the non-CAC group across all subgroups.

**Figure 1:**
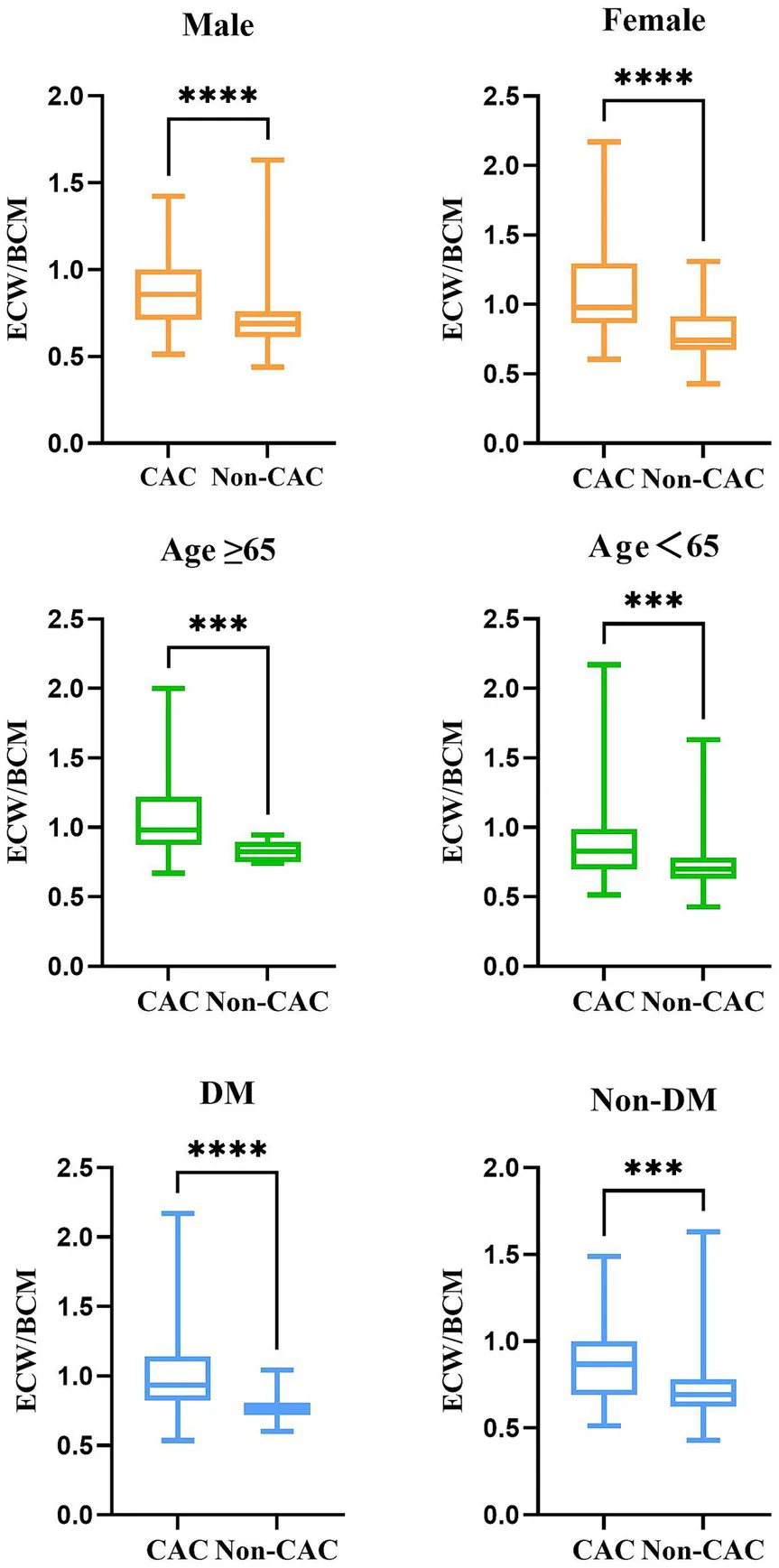
Subgroup analyses examining the association between ECW/BCM and CAC (***p < 0.001; ****p < 0.0001).

### Predictive value of ECW/BCM for CAC

ROC curve analysis was performed to assess the predictive value of ECW/BCM for CAC. The area under the ROC curve (AUC) of the baseline clinical model, which included age; sex; DM; heart disease; albumin, calcium and phosphorus levels; and use of vitamin D receptor activator was 0.847 (95%CI 0.791–0.903). After adding ECW/BCM to the baseline model, the AUC increased to 0.869 (95%CI 0.818–0.920, *p* = 0.058) (Figure 2). To further evaluate the incremental value of ECW/BCM, we carried out reclassification analyses. Adding ECW/BCM to the basic clinical model significantly improved continuous NRI (0.454, 95% CI: 0.166–0.751, *p* = 0.002) and IDI (0.04, 95% CI: 0.013–0.068, *p* = 0.004). These findings indicate that adding ECW/BCM significantly improved risk stratification and discrimination ability for identifying patients with CAC, despite the marginally non-significant elevation in AUC.

**Figure 2:**
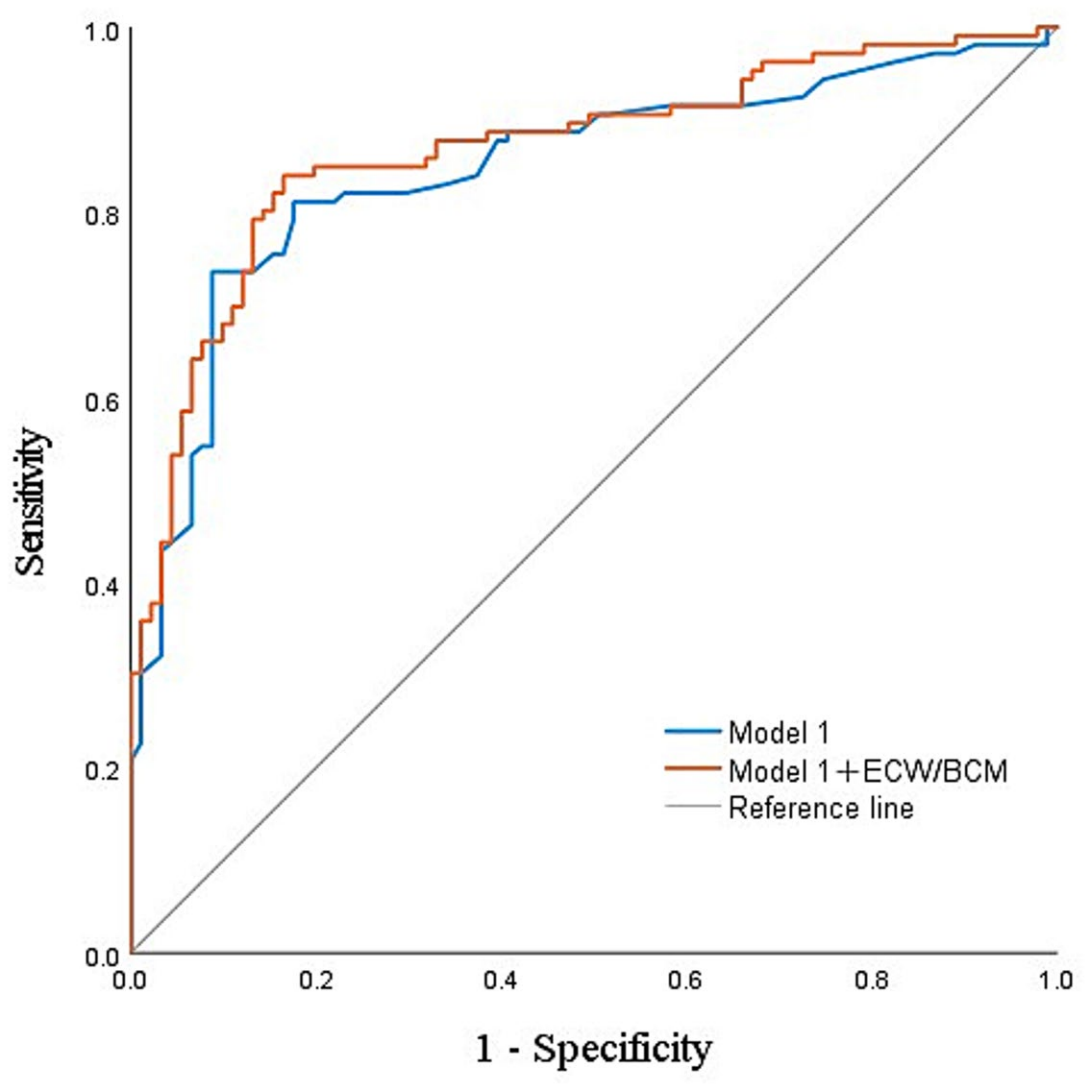
Receiver operating characteristic analysis for the presence of coronary artery calcification. Model 1 adjusted for age; sex; diabetes mellitus; heart disease; albumin; calcium; phosphorus; use of vitamin D receptor activator.

### Sensitivity analyses

For sensitivity analyses, we further analyzed the association between ECW/BCM and CACS. In univariate and multivariate analyses, ECW/BCM was statistically significantly associated with log-transformed CACS (model 3 in Supplementary Table S1; per 0.1 increase, *β* = 0.08, 95% CI, 0.023–0.136, *p* = 0.006).

## Discussion

To our knowledge, this is the first study to discuss the association between ECW/BCM and CAC in incident PD patients. We found that an increased ECW/BCM ratio was associated with a high presence of CAC, and similar results were observed in the subgroup analysis. The association remained statistically significant after extensive adjustment for confounding factors.

Coronary artery calcification score (CACS), as assessed by computed tomography, has been recommended for evaluating vascular calcification and is an independent predictor of all-cause mortality and cardiovascular events in chronic kidney disease (CKD) and dialysis patients (12, 13). CAC is common occurrence in dialysis patients. A study from Italy reported the CAC prevalence of 87% in hemodialysis (HD) patients (14), whereas the prevalence reported in the United States ranged from 25 to 66% (15, 16). In addition, Shang et al. (17) reported a prevalence of 75.8% in Chinese PD patients. In this study, we reported the prevalence of CAC at 53.81% in the PD patients, which was similar to the CDCS study (54.4%) (18). Therefore, early identification of risk factors is crucial for CAC in PD patients.

Body composition analysis via BIA have emerged as a valuable tool for the assessment of fluid volume and nutritional status in maintenance dialysis patient (19). BIA-guided fluid management exerts positive effects on blood pressure, arterial stiffness and survival in dialysis patients (20, 21). ECW is a direct indicator of extracellular fluid volume in the body and BCM represents the total mass of all metabolically active cells in the body. ECW/BCM is a mixed index of fluid overload and malnutrition. The ECW/BCM have received increasing attention in recent years. This ratio has been reported to be a sensitive indicator for nutritional assessment in patients with cancer, chronic radiation enteritis and anorexia nervosa (22–24). Compared with other markers of volume status, ECW/BCM performed better in assessing hydration status in AKI patients (25). Recent researchs have also revealed that ECW/BCM is a risk factor for intradialytic hypotension and functional impairment in HD patients (26, 27).

Volume overload activates the renin-angiotensin-aldosterone system, which in turn promotes the development of atherosclerosis by mediating endothelial dysfunction, oxidative stress, inflammatory responses, and smooth muscle cell proliferation via angiotensin II (28). In addition, the persistent volume overload leads to hemodynamic disturbances, resulting in endothelial injury and subsequently contributing to vascular calcification (6, 29). The close association between malnutrition, elevated CRP, and atherosclerosis has been well established, and their coexistence is referred to as the malnutrition, inflammation, and atherosclerosis/calcification (MIA) syndrome. We also found that patients in the CAC group had lower albumin and LTI (data not shown). Several studies have reported that inflammatory markers were significantly increased in the high-volume group (30, 31). Accordingly, we observed that patients in higher ECW/BCM groups had higher CRP. Our data also showed that albumin, creatinine, urea nitrogen, and LTI decreased with increasing tertiles of ECW/ BCM, suggesting that higher ECW/BCM reflects poorer nutritional status, which is similar to previous data in dialysis patients (32). Furthermore, we also found that patients in the high ECW/BCM group exhibited lower LTI and higher FTI, a body composition phenotype known as sarcopenic obesity, which is closely associated with patient survival and vascular calcification (33, 34).

In this study, we have analyzed the factors associated with ECW/BCM. We found that age was positively correlated with ECW/BCM, which is consistent with previous report showing that the normal value of this ratio varies with age (10). In addition, we noted that patients with higher ECW/BCM had a higher prevalence of diabetes, which may be attributed to the poorer nutritional status typically seen in diabetic patients. Interestingly, we found that males had lower ECW/BCM than females (0.76 [0.67, 0.92] vs. 0.86 [0.70, 1.04], *p* = 0.007), which has never been reported before. Further analysis showed no significant differences between the two groups in age, diabetes, albumin and CRP. Further studies are needed to clarify this relationship.

There are some limitations of this study. First, this was a single-center retrospective study with a relatively small sample size, which may have introduced selection bias, limited the external validity of our findings, and reduced statistical power to detect a significant improvement in AUC (*p* = 0.058). Second, due to the cross-sectional design of this study, causal relationships between ECW/BCM and CAC cannot be established. Third, several determinants of vascular calcification were not included in our analysis, which may result in residual confounding and affect the interpretation of the observed association. Fourth, despite extensive evidence supporting the general reliability and reproducibility of body composition analysis, our specific measurements may still be susceptible to systematic error and certain biases. Therefore, larger-scale, multicenter prospective longitudinal studies are needed to further validate our findings.

## Conclusion

In summary, ECW/BCM was significantly and independently associated with CAC in patients initiating PD. This finding may help identify a potential target for future research to investigate whether interventions targeting ECW/BCM could influence CAC progression.

## Data Availability

The raw data supporting the conclusions of this article will be made available by the authors, without undue reservation.
