# Supplementary material for: Ratio of extracellular water and body cell mass is associated with coronary artery calcification in peritoneal dialysis patients
Source: Front Med (Lausanne). 2026 Sep 21;13:1895552. doi: 10.3389/fmed.2026.1895552 (PMC13634810; doi:10.3389/fmed.2026.1895552)
Supplement: Supplementary file 1 [file Table_1.DOCX]

Table S1. Unadjusted and adjusted linear regression analyses of ECW/BCM for the coronary artery calcium scores*

|  | β (95% CI) | P value |
| --- | --- | --- |
| ECW/BCM(per 0.1 increase) | 0.204(0.144-0.264) | <0.001 |
| Model 1 | 0.073(0.017-0.130) | 0.011 |
| Model 2 | 0.068(0.010-0.127) | 0.023 |
| Model 3 | 0.080(0.023-0.136) | 0.006 |

** Log transformed coronary artery calcium scores

Model 1 adjusted for age, gender, history of DM and heart disease; Model 2 adjusted for all factors in model 1 plus albumin, cholesterol, calcium and phosphorus; Model 3 adjusted for all factors in model 2 plus use of Vitamin D receptor activator and Statin.
